# War and Disease. II.—The Russo-Japanese and South African Campaigns

**Published:** 1915-01-30

**Authors:** 


### WAR AND DISEASE.*

### The Russo-Japanese and South African Campaigns.

(Being the substance of the second of three Chadwick Trust lectures delivered by Col. F. M. Sandwith, M.D., Chairman of the British Red Cross Society, at the Royal Society of Arts on January 22.)

Colonel Sandwith said the officers of the Japanese Army considered that there were three grades of illness: (1) wounds or disease caused by hardships and the exposure incidental to warfare; (2) diseases, accidents, or injuries to which the soldier might succumb in circumstances beyond his control; (3) diseases due to disobedience to orders, immorality, eating unwholesome food, drinking dirty water, or other non-compliance with the rules of health laid down. The victims to the last of these received no sympathy. The way in which the Japanese avoided serious epidemics in their war with Russia was largely due to the men having been made to understand how great a factor in success was their attention to personal hygiene. This lesson had not been lost on the medical authorities of this country, and the French admired the British soldier for his hygienic observances. The chief aim of the Army medical officer was to maintain the soldier in clean surroundings, for conditions brought about by dirt might defeat all other precautions.

### The Modder River Camp.

No disease, for instance, dogged the footsteps of an army so much as typhoid; the French called it "manœuvre fever." For many years now it had been regarded as entirely a water-borne disease; several outbreaks in civil life had been traced to a polluted water supply. Of late, however, other causes had been proved, such as contamination of food by dust and by flies when the water supply was good and pure. In the South African War, Lord Methuen's force of 13,500 men was camped on the Modder River, on a level sandy plain. Each man dug his own sanitary trench, and in that way a large area of soil was contaminated. The winds covered the food with grit, flies multiplied rapidly, and were a source of great annoyance. The first case of typhoid was recognised on December 18, 1900, and by the end of that month eighteen cases of it had been admitted to hospital. The Guards suffered most severely, and the Highland Brigade least, the reason being that the camp of the latter lay where it was protected from the wind, which was not the case with the Guards. The interior of the tents and the food were black with flies. Many who had recovered from typhoid harboured and excreted the bacilli long after their symptoms had ceased; and such typhoid-carriers were especially dangerous under war conditions, owing to the close proximity of the men to each other, and the necessity for wearing their clothes for a long time. He described the course of a typhoid attack, and remarked that the death-rate varied between 5 per cent. and 20 per cent.

Owing to the difficulty of recognising the disease during war time, the mortality was higher in troops on active service, especially as the British soldier was diffident about reporting himself "sick." In the South African campaign there were 8,022 deaths from the disease, 19,454 were invalided, and 30,208 were either returned to duty or otherwise discharged. Each case, on an average, was fifty-six days under treatment, and each patient was, necessarily, a focus of danger during that period.

### Conditions at Ladysmith.

During that war we had an army chiefly consisting of young, susceptible material, unprotected by previous experience in campaigning, and ignorant as to how to protect themselves against the conditions in South Africa. They found themselves on an arid, treeless plain, very hot and dusty by day, and cold by night. The bad conditions were enhanced in Ladysmith, where the half-starved garrison had to draw its water supply from a river known to be polluted. During the 120 days of siege there were 1,280 cases of typhoid, with 360 deaths; and 1,841 cases of dysentery, with 105 deaths. The same disease attacked the troops which entered Bloemfontein, where the British found two churches filled with Boers suffering from typhoid. The difficulty of getting any water at all made all precautions in that site unavailing. Tents had to be pitched for the relief of about 130 men who collapsed from the effect of the heat. The Battle of Paardeburg was commenced when our men had been marching twenty-four hours; some had been able to snatch a hurried meal, but many had eaten nothing during that time. The fighting lasted twelve hours.

Cronje surrendered a week later, and the Boer laager was found to contain 160 sick and wounded, including ten cases of typhoid. The water intake of the Army lay down stream from this spot. The wells within the Orange Free State capital had previously become much contaminated, owing to the complete absence of drainage in the neighbourhood. Not until April 23 did General Ian Hamilton succeed in capturing the waterworks, which were then found to be out of order. Within ten days of the entry into Bloemfontein many cases of typhoid were in hospital; and during the twenty weeks from March 16 to July 27 there were 1,134 deaths in Bloemfontein, of which 964 were due to typhoid. There was now a means of circumventing the danger in anti-typhoid inoculation. It was satisfactory that the soldiers themselves had become more and more convinced of the efficacy of this measure, and that the vast majority of the soldiers now on active service were so protected. Typhoid fever is found to be five or six times as common in non-inoculated as in the inoculated. In Avignon, during the summer of 1912, typhoid fever broke

* The first lecture was reported last week.

out in barracks containing 2,053 men, 1,366 of whom were protected. Among the 687 unprotected troops there were 155 cases of typhoid, of whom twenty-one died, while there was not one case among the protected. In Canada, among the camps of the Canadian Pacific Railway, of 5,500 men protected there were only two cases of typhoid; while among 4,500 non-protected there were 228 cases. In face of such facts it was difficult to understand the attitude of those who tried to persuade the soldier to refuse the treatment. Dr. Sandwith proceeded to argue in favour of compulsory prophylactic inoculations, and quoted Major Russell's communication to the *British Medical Journal*, pointing out the good result in 20,000 men —viz., not more than 1 in 90 even moderately ill. A paragraph in the *Times* a few days ago showed that the value of vaccination against typhoid had been proved beyond doubt during the five months of the war. The British Army now was not suffering extensively from typhoid, and he wished he could say the same for the Belgian troops.

### Dysentery in Past Wars.

The lecturer then proceeded to discuss dysentery and cholera. Since the Peloponnesian War there had scarcely been a protracted military campaign in which dysentery had not scourged the contending armies. In the American Civil War 30 per cent. of the total deaths among the Federal troops were due to dysentery. In the Franco-Prussian War there were 38,652 cases and 2,380 deaths from dysentery in the German Army, and the troops, on their return home, spread the infection in many districts, some of which were not yet free from it. In South Africa there were 38,108 cases of dysentery. The great means of preventing an epidemic of dysentery was to isolate victims until they ceased to be carriers. Attempts were now being made to discover an anti-dysenteric substance.

### Cholera in Fighting Armies.

Cholera was the most deadly of the group of diseases now being considered. On October 22 last ninety-eight cases of cholera were reported in Austria and 231 in Hungary. The disease has also appeared in some of the Russian towns. Dr. Fleming and others had returned from the Russian theatre of war, where they had been investigating the kind of cholera bacillus present, and were probably now engaged in preparing an anti-toxin for use should our own troops develop the disease. The average death-rate in cholera was 50 per cent. The water supply was the chief source of contagion. Among the Egyptian force led by Lord Kitchener in 1896, death in those attacked occurred very suddenly. Dr. Sandwith sketched the course of various cholera epidemics in fighting armies, and showed what considerable immunity resulted among half-a-million Greeks who were protected against cholera. The authorities responsible for the health of our troops were now preparing against the possibility of the invasion of this deadly enemy.

A number of very instructive pictures, including photomicrographs of bacilli, were, at the conclusion of the lecture, exhibited on the screen.

### Education of the Soldier.

Sir Frederick Treves, in thanking Dr. Sandwith in the name of the audience, emphasised what he said as to the attitude to be adopted towards typhoid fever in war. The first line of attack was the education of the soldier. He was in South Africa during the war there, and in Japan when that country was fighting against Russia, and he was much struck by the difference between the soldier who had been told (the Japanese) and the soldier who had not been told (the British). The education of the British soldier in these matters was now very thorough.

He added that the second line of attack was the sanitary condition of the Army, and in the present campaign our sanitary arrangements were unprecedented, though they were little talked about. But a further precaution was needed (as shown by even medical officers contracting the disease)—namely, inoculation. In the British Expeditionary Force, since the war began, there had been only 212 cases of typhoid, and 201 of these were in unprotected men. Of the 201, 173 had not been inoculated at all; the remaining twenty-eight had either only had one inoculation or had not been injected for over two years. Only eleven of the 201 had been fully inoculated; twenty-two deaths occurred out of the 212 cases, all in uninoculated men. There had been no death among the inoculated. In regard to transport, there had never been anything so revolutionary in field service work as in the alteration in the mode of transport carried out in the present war, especially in the matter of ambulance trains and motor ambulances. Right at the Front there was a motor laboratory. It was defective transport which killed the men; but it would not be possible to dispense with the old horse ambulance at the firing line.

Hearty thanks were accorded to Dr. Sandwith for his lecture.

